# Suspected Regional Lymph Node Metastasis in Hepatic Alveolar Echinococcosis: A Case Report

**Published:** 2020

**Authors:** Qiang WANG, Yu CUI, Li REN, Haijiu WANG, Zhixin WANG, Hu WANG, Haining FAN

**Affiliations:** 1. Department of Hepatobiliary and Pancreatic Surgery, Affiliated Hospital of Qinghai University, Xining, Qinghai 810001, P.R.China; 2. Qinghai Province Key Laboratory of Hydatid Disease Research, Xining, Qinghai 810001, P.R.China; 3. Health Commission of Qinghai Province, Xining, Qinghai 810001, P.R.China

**Keywords:** Hepatic alveolar echinococcosis, Regional lymph node metastasis, China

## Abstract

There is no direct evidence to support the existence of regional lymph node metastatic routes in hepatic alveolar echinococcosis, and only a few literature have been reported. There was a case of hepatic alveolar echinococcosis suspected of metastasis of regional lymph node in Clinical Center for Hydatidosis, Qinghai Province, China. The patient was a 24-yr-old male from pastoral area of Seda County, Sichuan Province, China in 2018. He was admitted to the hospital for physical examination and had no special discomfort. Preoperative examination showed liver occupancy and regional lymph node enlargement in the space between liver and stomach. Hepatectomy and lymph node resections were performed. Postoperative pathological results showed that both primary and metastatic lesion were of alveolar echinococcosis. Recovery of patient was good without complications and recurrence. In this case, metastasis was considered because the liver lesion was not directly connected to the lymph node. However, the case was still suspected due to the lack of pathological examination of other lymph nodes in the lymphatic return pathway. Regional lymph node metastasis may be one of the metastatic ways of alveolar echinococcosis.

## Introduction

Alveolar echinococcosis is a kind of zoonotic disease, which is prevalent in pastoral areas. Although it grows slowly and belongs to benign diseases, it could transfer from liver to other organs (1,2). According to current reports, the main ways of metastasis include “proximal infiltration, distant hematogenous/lymphatic metastasis”. However, there is no conclusive evidence to support the regional lymph node metastasis pathway (3).

K. Buttenschoen et al. (3) reported a case of hepatic alveolar echinococcosis with possible regional lymph node metastasis in 2009. In addition, few cases have been reported so far. One case of suspected regional lymph node metastasis of hepatic alveolar echinococcosis was treated in our center.

## Case Presentation

Written informed consent was obtained from patient and the article was approved by the Ethics Committee of the Affiliated Hospital of Qinghai University.

The patient was a 24-yr-old male from pastoral area of Seda County, Sichuan Province, China in 2018. He was admitted to the hospital for physical examination, and had no special discomfort. Except for the slight increase of direct bilirubin, the other indicators included blood routine, coagulation and renal function were within normal ranges.

**Fig. 1:**
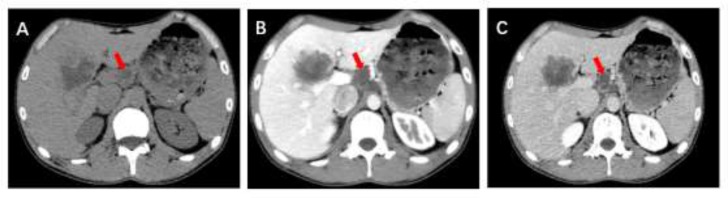
CT plain and enhanced scan. (A) Hydatid lesion in liver and enlarged lymph node in the hepatogastric space with diameter of 2.13 cm. B (portal vein phase) and C (delayed phase) showed circular enhancement.

**Fig. 2:**
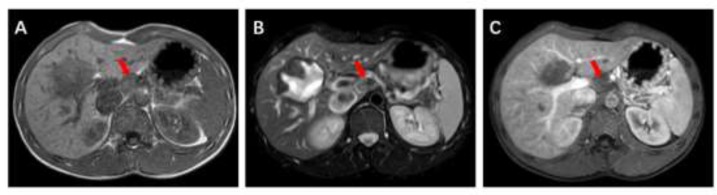
MRI scan. A (T1), B (T2) Sequence and C (enhanced scan) showed enlarged lymph node in hepatogastric space with slightly longer T2 signal.

The abdominal computed tomography (CT) showed lesion located in S4-5-6 and slightly low-density lymph node existed in the hepatogastric space with diameter of 2.13 cm (the lesion on maximum diameter was not displayed in order to show the lymph node better, Fig. 1. A, B and C). Branches of right hepatic artery and portal vein were close to the lesion. It was suspected of hepatic alveolar echinococcosis (P2N1M0, based on expert consensus of alveolar echinococcosis (4)) with regional lymph node metastasis. In addition, there were several cysts in the liver. No special findings were found in the lung and brain scans. The results of magnetic resonance imaging(MRI) scan also considered hepatic alveolar echinococcosis (Fig. 2. A, B and C).

Hepatic alveolar echinococcosis is a space-occupying disease of the liver. It is necessary to differentiate it from benign diseases such as hepatic cyst, hemangioma, lipoma, bacterial liver abscess, and malignant diseases such as primary hepatocellular carcinoma, secondary hepatocellular carcinoma and sarcoma.

**Fig. 3:**
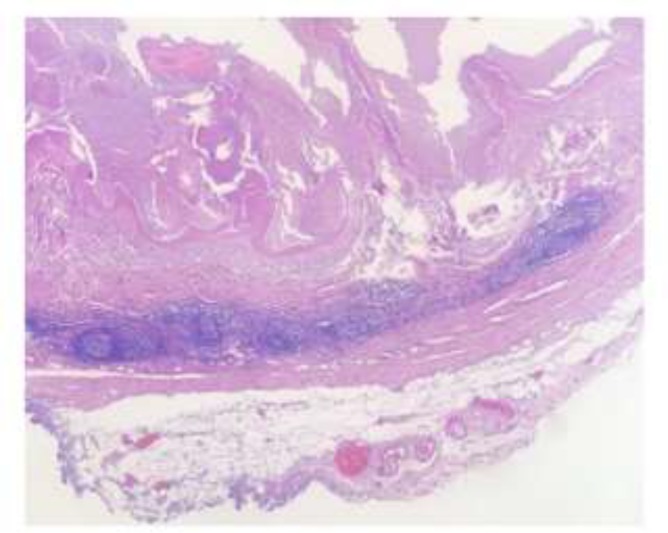
Postoperative microscopic findings. HE staining of lymph node, ×10, showed that the central structure of lymph node was basically all hydatid tissue, and a small number of follicles under the capsule

Excluding surgical contraindication, hepatectomy and lymph node resections were performed on May 10, 2018. During the operation, we found that the primary focus adhered to the base of gallbladder and the size was about 7.5 *7.0 cm. The lymph node in the hepatogastric space was cystic and contained gray yellow chyle-like substance with wall thickness of about 0.2 cm. Postoperative pathology confirmed that both primary focus and regional lymph node were alveolar echinococcosis. (Fig. 3).

The follow-up period began after out of hospital. The patient recovered well without complications. After operation, the patient took albendazole regularly for half a year, and no recurrence was found.

## Discussion

Lymph node metastasis is one of the possible metastatic ways of hepatic alveolar echinococcosis, but there is no direct evidence to support it at present. Now the mainstream view is that germinal cells may play an important role in metastasis, because they can become various kinds of “hydatid cells” through proliferation and differentiation, and eventually form a metastatic lesion of hydatid disease (5,6).

However, could germinal cells or “hydatid cells” really metastasize to lymph nodes? How to transfer? Matsuhisa (7) believes that hydatid lesions can be transferred to other organs after invading intrahepatic veins. It seems possible for metastasis of lung and brain, because metastatic lesions are far from the primary focus, and blood transmission can provide power that “hydatid cell” metastasis needs. However, for regional lymph nodes, “hydatid cells” or germinal cells first need to reach the systemic blood circulation through the intrahepatic venous sinus, and then enter the lymph nodes at a certain opportunity, which seems unlikely (3). “Hydatid cells” or germinal cells are transferred to regional lymph nodes through lymphatic reflux (3). Suspicious metastasis of peripancreatic lymph node in hepatic alveolar echinococcosis was already reported (8).

In this case, the focus of the liver is not directly connected with the regional lymph node in the hepatogastric space, which excludes the possibility of infiltration, and it should be considered as regional lymphatic metastasis. However, the case is still suspicious because no comprehensive and systematic histological verification of all the lymph nodes in lymphatic return pathway has been carried out. We support K. Buttenschoen’s view on the route of infection of regional lymph nodes with hepatic alveolar echinococcosis. However, there is no histological confirmation of relevant lymph nodes, it is a little inappropriate to conclude “there is regional lymph node metastasis pathway of alveolar echinococcosis”, and further research is needed.

## Conclusion

It is very important to explore the presence of regional lymph node metastasis of alveolar echinococcosis in order to reduce the recurrence rate. Combining with the published literature and a case in our center, we suggest that regional lymph node metastasis may be one of the ways of alveolar echinococcosis metastasis. We need more convincing cases and research.
